# Deubiquitylases in developmental ubiquitin signaling and congenital diseases

**DOI:** 10.1038/s41418-020-00697-5

**Published:** 2020-12-17

**Authors:** Mohammed A. Basar, David B. Beck, Achim Werner

**Affiliations:** 1 Stem Cell Biochemistry Unit, National Institute of Dental and Craniofacial Research, National Institutes of Health, Bethesda, MD 20892 USA; 2 Metabolic, Cardiovascular and Inflammatory Disease Genomics Branch, National Human Genome Research Institute, National Institutes of Health, Bethesda, MD 20892 USA

**Keywords:** Deubiquitylating enzymes, Disease genetics, Neurological disorders

## Abstract

Metazoan development from a one-cell zygote to a fully formed organism requires complex cellular differentiation and communication pathways. To coordinate these processes, embryos frequently encode signaling information with the small protein modifier ubiquitin, which is typically attached to lysine residues within substrates. During ubiquitin signaling, a three-step enzymatic cascade modifies specific substrates with topologically unique ubiquitin modifications, which mediate changes in the substrate’s stability, activity, localization, or interacting proteins. Ubiquitin signaling is critically regulated by deubiquitylases (DUBs), a class of ~100 human enzymes that oppose the conjugation of ubiquitin. DUBs control many essential cellular functions and various aspects of human physiology and development. Recent genetic studies have identified mutations in several DUBs that cause developmental disorders. Here we review principles controlling DUB activity and substrate recruitment that allow these enzymes to regulate ubiquitin signaling during development. We summarize key mechanisms of how DUBs control embryonic and postnatal differentiation processes, highlight developmental disorders that are caused by mutations in particular DUB members, and describe our current understanding of how these mutations disrupt development. Finally, we discuss how emerging tools from human disease genetics will enable the identification and study of novel congenital disease-causing DUBs.

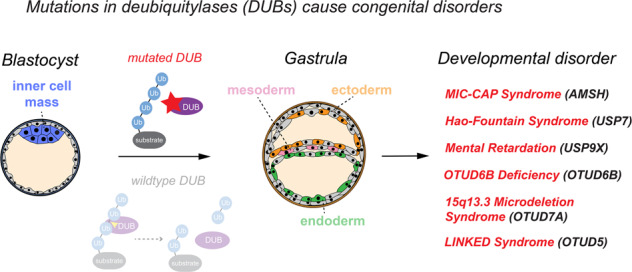

## Facts

- Deubiquitylases (DUBs) are a class of ~100 human enzymes that regulate ubiquitin signaling by processing ubiquitin precursors, hydrolyzing ubiquitin chains, and cleaving ubiquitin modifications from substrates.
- Intricate regulatory mechanisms ensure spatial and temporal regulation of DUB activity and substrate recruitment to allow DUBs to integrate signals during development and coordinate developmental cell-fate decision.
- DUBs regulate gene expression (through deubiquitylating histones and modulating the stability of chromatin regulators/transcription factors) and signaling pathways to control metazoan development.
- Mutations in particular DUBs cause developmental disorders, but the molecular mechanisms and cognate substrates or E3 ligases are often unknown.
- Many DUBs are intolerant to haploinsufficiency and missense mutations in the general human population, suggesting that their dysregulation likely causes developmental diseases.

## Open questions

- How are specific DUBs regulated during embryonic and postnatal development to achieve their functions in cell-fate determination?
- What are the mechanisms and substrates of DUBs whose mutations underlie developmental diseases?
- Which other DUBs cause developmental diseases? Can we utilize tools from human genetics to identify these DUBs and study their (patho-)physiological functions and mechanisms?

## Introduction: the ubiquitin code and DUBs in early development

**Fig. 1:**
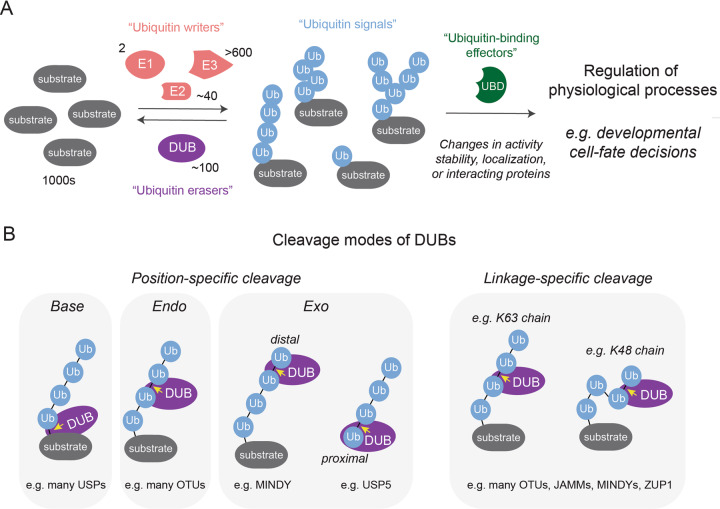
Overview of how ubiquitin signaling regulates developmental cell-fate decisions and cleavage modes of DUBs. **A** To initiate ubiquitin signaling, an enzymatic cascade, consisting of ubiquitin E1 activating, E2 conjugating, and E3 ligating enzymes, decorates substrates with topologically different ubiquitin modifications. Effector proteins containing various ubiquitin-binding domains (UBDs) interpret the ubiquitin signals and mediate changes in the substrate activity, stability, localization or interacting proteins. This controls cellular behavior during many physiological processes, including development. DUBs are important regulators of this ubiquitin code by reversing ubiquitin modifications, thus modulating or terminating signaling. **B** Cartoons depicting different position- and linkage-specific cleavage modes by which DUBs can act on their substrates. The yellow arrow indicates the peptide/isopeptide bond that is hydrolyzed in each example.

During metazoan development, stem cells of the embryo undergo self-renewal, commit to differentiation programs, and produce and react to signaling molecules to ensure proper formation of specialized cell types, tissues, and organs. The precise execution of these processes is often controlled by ubiquitylation, an essential posttranslational modification (PTM) that regulates the stability, activity, localization, or interaction landscape of substrates [1–3]. The differential outcomes of ubiquitylation are accomplished by elaborate enzymatic cascades that synthesize ubiquitin signals, which are covalently linked to substrates and recognized and interpreted by various effector proteins (Fig. 1A) [4–6]. Research in recent decades has elucidated key principles of this ubiquitin code [4]. Substrates can either be modified with ubiquitin monomers or with structurally distinct ubiquitin polymers that are linked via the N terminus or one of the seven internal lysine residues (K6, K11, K27, K29, K33, K48, K63). These ubiquitin polymers can be homotypic (chains with only one linkage type) or heterotypic (chains with at least 2 different linkage types) [7]. Mono- or multi-monoubiquitylation of substrates often result in changes in the interaction landscape of the modified protein and play important roles during e.g., transcription, translation, and endosomal sorting [8–11]. Modification of substrates with homotypic and heterotypic ubiquitin polymers elicits various downstream effects, which depend on the linkage type(s) and architecture of the ubiquitin chain. Well established examples with relevance to this review include homotypic K11- or K48-linked chains that mediate degradation via the 26S proteasome [12, 13], homotypic M1- or K63-linked chains that allow for formation of signaling complexes during NF-kB activation [14, 15] and DNA repair [16–18], and homotypic K63-linked chains that mediate autophagic degradation of protein complexes, aggregates, and damaged organelles [19].

Ubiquitylation regulates biological pathways in a highly specific and reversible manner, which enables ubiquitin signaling to control cellular behavior and decision-making during embryonic development [1, 3]. Specificity is achieved by more than 600 ubiquitin E3 ligases, which bind distinct sets of substrates and cooperate with 2 E1 and ~40 E2 enzymes to mediate transfer of ubiquitin monomers or chains to thousands of cellular substrates [4, 20, 21]. Reversibility is ensured by ~100 human deubiquitylases (DUBs), a family of enzymes that processes ubiquitin precursors, edits chain architecture, or cleaves ubiquitin signals from substrates. Through these activities, DUBs maintain a functional ubiquitin pool for conjugation and modulate or terminate signaling responses [1, 22–24].

DUBs can deubiquitylate a broad range of substrates in fundamental cellular processes including transcription, translation, cell cycle progression, vesicular trafficking, autophagy, proteasomal degradation, and intracellular signaling to control various aspects of stem cell maintenance, differentiation, and development [1, 3, 25–30]. Consistent with these essential functions, genetic deletion of a number of DUBs are embryonic, early postnatal, or perinatal lethal in mice (examples discussed in this review include *USP7* [31], *USP8* [32], *USP9X* [33, 34], *USP16* [35], *USP22* [36], *BAP1* [37], *OTUB1* [38], *AMSH* [39], *OTUD6B* [40], and *OTUD5* [41, 42]) and kockdown of tens of DUBs has been shown to be lethal or to cause severe defects during zebrafish development [43, 44]. It is therefore not surprising that dysregulation of DUBs is linked to many human diseases, including cancer, neurodegeneration, and inflammatory syndromes [1, 25–28, 45–47]. A growing number of studies has also implicated aberrant activities of several DUBs as drivers of distinct congenital diseases (Table 1), providing further evidence for essential roles for these enzymes in controlling ubiquitin signaling during embryonic and postnatal development.

**Table 1:** DUBs whose mutations underlie developmental diseases.

Gene	Disease	Phenotypes	Mutations	Proposed molecular disease mechanism/substrates
*AMSH*	MIC-CAP syndrome (*MIM:614261*)	Microcephaly with progressive cortical atrophy, intractable epilepsy, profound developmental delay, and multiple small capillary malformations on the skin [146]	Loss-of-function; recessive mutations that reduce levels, protein interactions, and, catalytic activity [146, 180–183]	Increased RAS and PI3K signaling through dysregulation of endosomal sorting [146]
*USP7*	Hao-Fountain syndrome *(MIM:616863)*	Seizures, behavioral abnormalities, hypogonadism, and hypotonia [141]	Loss-of-function; heterozygous deletions or nonsense mutations [141]	Impaired endosomal protein recycling and actin dynamics through loss of USP7-mediated regulation of MAGE-L2-TRIM27 and WASH [141]
*USP9X*	Mental retardation, X-linked 99 *(MIM: 300919*)	Global developmental delay, intellectual disability, brain abnormalities, hypotonia, motor and speech delay [195, 199]	Loss-of-function; hemizygous mutations [195, 199]	Decreased TGF-β signaling (SMAD4/SMURF1) [199]; impaired centriole duplication (STIL) and cilia formation (NPHP5) [201, 204]; impaired dendritic spine formation and maintenance (ankyrin-G) [144, 145]
*USP9X*	Mental retardation, X-linked 99, syndromic *(MIM: 300968)*	Female-restricted, intellectual disability associated with characteristic facial features, short stature, cardiac, and structural brain abnormalities [197, 198]	Loss-of-function; heterozygous missense and nonsense mutations [197, 198]	Decreased TGF-β signaling (SMAD4/SMURF1) [199]; impaired centriole duplication (STIL) and cilia formation (NPHP5) [201, 204]; impaired dendritic spine formation and maintenance (ankyrin-G) [144, 145]
*OTUD5*	LINKED syndrome	Global developmental delay, intellectual disability, central nervous system, craniofacial, cardiac, skeletal, and genitourinary anomalies [41]	Loss-of-function; hemizygous deletion and missense mutations that affect protein levels, localization, and catalytic activity [41]	Impaired chromatin remodeling at neuroectodermal enhancers due to aberrant degradation of chromatin regulators (e.g., ARID1A/B, HDAC2, HCF1) [41]
*OTUD6B*	Multiple congenital anomaly disorder *(MIM:617452)*	Global developmental delay, feeding difficulties, structural brain abnormalities, and congenital heart disease [40, 214]	Loss-of-function; recessive nonsense and missense mutations [40, 214]	Unclear; possible functions in protein translation [215] and proteasome assembly [40]
*OTUD7A*	15q13.3 microdeletion syndrome *(MIM:612001)*	Intellectual disability, seizures, language impairment, dysmorphic features, neuropsychiatric disorders [216–219]	Loss-of-function; deletion mutations [216–219], one report of biallelic missense mutation [221]	Unclear; possible functions in proteasome assembly [221] and DNA damage responses [235]
*USP27X*	Mental retardation, X-linked 105 *(MIM: 300984)*	Intellectual disability, language impairment, behavioral problems [236]	Loss-of-function; hemizygous nonsense and missense mutations [236]	Unclear; poorly characterized histone-directed DUB [107]
*ALG13*	Epileptic encephalopathy *(MIM: 300884)*	Seizures, delayed psychomotor development, dysmorphic features [237, 238]	Loss-of-function; Hemizygous and heterozygous missense and deletion mutations [237]	Reduction in N-linked glycosylation due to reduction of UDP-GlcNAc transferase activity of ALG13 [237, 238]; unclear whether the OTU domain in ALG13 is active and functional

Table summarizing DUBs that have been shown to cause congenital disorders when mutated. Disease name, patient phenotypes, type of mutations, and the proposed molecular mechanism of pathogenesis are shown.

Here, we review structural, functional, and regulatory features of DUBs that allow this class of enzymes to fulfill their roles during development. We summarize general mechanisms of how DUBs regulate stem cell self-renewal and differentiation processes and our current understanding of how mutations in particular DUBs cause congenital diseases. Finally, we discuss how recently developed genetic resources can help identify candidate DUBs critical for development.

## Structural and functional features of DUBs

### DUB families

Since the discovery of DUBs in the mid-1980s [48–50], extensive studies have defined them as a structurally diverse set of ~100 human proteases that can be divided into two groups according to their enzymatic mechanisms. First, zinc-dependent JAB1/MPN/MOV34 (JAMM) metalloproteases (12 human members) and second papain-like cysteine proteases that, based on their catalytic domain, are further subclassified into six families: ubiquitin-specific proteases (USPs, 56 members), ovarian tumor proteases (OTUs, 17 human members), ubiquitin carboxy-terminal hydrolases (UCHs, 4 human members), the Machado–Joseph disease proteases (MJDs, 4 human members), and two more recently identified families, the motif interacting with ubiquitin-containing novel DUB family (MINDYs [51], 5 human members) and zinc finger containing ubiquitin peptidase 1 family (ZUP1, 1 human member [52–55]). Eleven of these 99 DUBs have lost critical catalytic residues and are thought to be catalytically inactive [56].

### DUBs can remove ubiquitin from substrates or cleave ubiquitin-linkages

Several in-depth discussions on DUB enzymology, structure, and substrate specificity have recently been published and we refer interested readers to these seminal reviews [22, 27, 57–60]. To provide the mechanistic framework for the role of DUBs in development, we highlight a few general structural and functional properties of DUBs.

To maintain free ubiquitin pools for conjugation and to regulate ubiquitin signaling, DUBs hydrolyze peptide or isopeptide bonds between ubiquitin and a substrate or within ubiquitin chains. In this process, DUBs generally utilize their catalytic domain to recognize and remove the distal (C-terminal glycine-contributing) ubiquitin from the proximal (lysine- or methionine-contributing) ubiquitin or the substrate. By utilizing additional ubiquitin-binding sites and/or substrate interaction motifs, DUBs have evolved specificities for cleavage at particular positions in the ubiquitin chain or linkage types [22, 27, 57–59] (Fig. 1B). For instance, many DUBs of the USP family encode substrate interaction motifs and cleave ubiquitin chains from substrates (base cleavage) [22], while other DUBs prefer to cleave ubiquitin chains from the middle (endo-cleavage, e.g., most OTU DUBs [61]) or from the distal or proximal end of the chain (distal *exo* cleavage, e.g., MINDY [51, 62], and proximal *exo*-cleavage, e.g., USP5 [63], respectively). In addition, some DUBs display exquisite linkage specificity (e.g., some members of the OTUs [61], JAMMs [64–66], MINDYs [51], ZUP1 [52–55] and the USPs USP30 [67–69] and CYLD [70, 71]) and only cleave one or a distinct set of ubiquitin-linkage types, while other DUBs (e.g., most USPs [72]) are more promiscuous.

## Regulatory principles impinging on DUBs

Ubiquitylation frequently orchestrates core signaling networks essential for stem cell maintenance and differentiation. During these processes, it is important that ubiquitin signals are tightly controlled. Similar to their E3-ligase counterparts [1, 3, 73, 74], DUBs are subject to a plethora of regulatory principles that impinge on their abundance, localization, activity, and substrate recruitment, thus allowing temporal and spatial regulation of deubiquitylation [26–28, 59, 75]. Here, we will briefly summarize key mechanisms of DUB regulation highlighting examples that are recent and have particular relevance for development.

### Regulation of DUB abundance

During development, similar to other signaling proteins, DUBs are commonly controlled at the level of their synthesis and degradation [11, 76–78]. For instance, two histone-directed DUBs, USP44 and USP22, are antagonistically regulated in their mRNA expression to ensure faithful stem cell differentiation [79–81]. In addition, transcription of other DUBs is upregulated at stages of differentiation or in specialized cell types when they are functionally required (e.g., ATXN3, UCHL1, and OTUD7A in neuronal cells and in the brain [77, 82–90]). Besides transcriptional control, DUBs are also frequently subject to regulated ubiquitin-dependent degradation. This process can be induced by stimulus-dependent proteolytic processing (as e.g., shown for CYLD [91, 92], A20 [93], and USP1 [94, 95]) and some DUBs can counteract their own degradation via auto-deubiquitylation. For example, phosphorylation of USP4 by AKT activates and thus stabilizes this DUB, a process required for proper regulation of TGF-β signaling during embryonic development (further discussed below) [96, 97]. Taken together, transcriptional and posttranslational mechanisms cooperate to enable adjustment of the cellular DUB repertoire required for a particular developmental process or tissue function.

### Regulation of DUB localization

Another frequent mode of regulation in eukaryotic cells is targeted localization [98]. Experiments in mammalian tissue culture cells analyzing GFP-tagged DUBs by fluorescence microscopy have revealed that at steady-state conditions, specific DUBs are localized to distinct sites such as the cytoplasm, nucleus, select organelles, or cellular membranes [99]. These subcellular localizations can be modulated through various mechanisms. First, a number of DUBs are expressed as multiple splice variants, which can encode domains that allow for isoform-specific subcellular localization and function. Examples include USP19 (cytosolic and ER [100, 101]), USP33 (ER and Golgi [102]), and USP35 (cytosolic, ER, and lipid droplets [103]). Second, several DUBs are shuttled between the nucleus and cytoplasm via reversible PTMs [96, 97, 104, 105]. For instance, AKT-mediated phosphorylation relocates nuclear USP4 to the cytoplasm and membranes to regulate TGFβ signaling during embryonic stem cell differentiation [96, 97]. In addition, UBE2O-mediated multi-monoubiquitylation of BAP1 sequesters this DUB to the cytoplasm during adipocyte differentiation [105]. Third, many DUBs are recruited to their substrates with the help of adapter proteins. This regulatory concept is frequently applied by histone-directed DUBs. For example, USP44 (through the N-CoR complex [106]), USP51, USP27X, and USP22 (through ATXN7L3 and ENY2 [107]), and BAP1 (through FOXK1/2 and ASXL1/2/3 [37, 108, 109]) are recruited to specific regions on chromatin, where they counteract monoubiquitylation of H2A and H2B to regulate gene expression changes required for various aspects of stem cell maintenance and differentiation (see below). In addition, DUB recruitment to substrates via adapter proteins can also be utilized to stabilize transcription factors. This is exemplified by USP7, which has recently been shown to be targeted to stemness factors SOX2, NANOG, and OCT4 via BACH1 to counteract their degradative ubiquitylation, thus ensuring hESC self-renewal [110]. Taken together, various mechanisms control the dynamic localization of DUBs to enable spatial restriction of ubiquitin signaling during development.

### Regulation of DUB activity and substrate recruitment

In addition to control of abundance and localization, DUBs are also subject to regulation at the level of their activity and substrate recruitment (reviewed in detail in [22, 28, 59, 75]). We will briefly outline these principles in the following section by describing their relevance in ubiquitin signaling during developmental cell-fate decisions.

#### Regulation of DUB activity through interactions in cis or trans

Catalytic activities of DUBs can be modified through interaction with accessory domains or proteins. For instance, ubiquitin-binding and activity of BAP1, an essential, histone-directed DUB that regulates gene expression networks during development [37, 111], is stimulated by binding to the transcription regulators ASXL1, ASXL2, or ASXL3 [111–113]. This activation is critically controlled by monoubiquitylation of ASXL proteins [114]. In a different example, USP7, a DUB with pivotal roles in stem cell self-renewal and differentiation (see below), requires its C-terminal ubiquitin-like domains to fold onto the catalytic USP domain [115, 116] resulting in increased intrinsic USP7 activity, which can be further stimulated by binding of an interacting protein in the form of GMP-synthase [115, 117]. Similarly, the interaction of UAF1 and WD-repeat-containing proteins with specific DUBs regulates their catalytic activity [118–120]. One example is given by USP1, an important negative regulator of osteoblastic differentiation [121]. Such allosteric regulation is also well described for DUBs that are incorporated into large macromolecular complexes such as the proteasome [122–126] and the SAGA histone acetyltransferase complex [127–129] (i.e., USP22, an essential regulator of stem cell differentiation [36, 81]). Finally, also self-association of DUBs has been shown to regulate DUB activity. USP25, a regulator of the WNT signaling pathway [130], forms active dimers and autoinhibited tetramers in vitro and in cells [131–133]. Thus, various types of interactions in *cis* or *trans* can activate or inhibit the activity of DUBs that regulate important aspects of differentiation. However, in most cases, how these mechanisms are implemented to control ubiquitin signaling during embryonic and postnatal development remain unclear and will require further investigation.

#### DUB interactions with E3s

In addition to interacting with allosteric regulators, DUBs also frequently associate with ubiquitin E3 ligases in cells [134]. This coupling of opposing enzymatic activities has emerged as a functionally important feature that can regulate ubiquitin signaling in diverse and complex ways [135]. DUB-E3 interactions are used for mutual ubiquitin-dependent regulation (e.g., to control each other’s stability, see above) or for editing ubiquitin chain architecture on particular substrates (as shown for the hybrid DUB/E3 enzyme A20 and CYLD-ITCH complexes during inflammatory signaling [136, 137]). Moreover, DUB-E3 complexes can work in direct opposition on shared substrates, thus fine-tuning responses during cell-fate decisions. For instance, USP9X associates with the ubiquitin E3-ligase WWP1 to modulate DVL2 ubiquitylation to specify canonical and noncanonical responses of WNT signaling, which controls various aspects of stem cell self-renewal and differentiation [138–140]. Similarly, USP7, an integral part of the ubiquitin E3-ligase complex MAGE-L2-TRIM27, acts as a molecular rheostat to control the activity of the actin nucleating protein WASH during neurodevelopment [141] (further discussed below), illustrating the exquisite regulation afforded by coupling opposing DUB and E3 activity during differentiation processes.

#### Regulation by PTMs

**Fig. 2:**
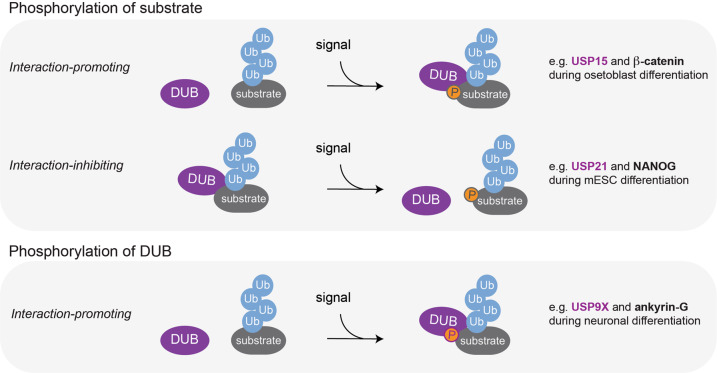
Stimulus-induced phosphorylation regulates DUB-substrate recruitment during differentiation. Stimulus-induced phosphorylation is frequently used to regulate DUB-substrate recruitment during developmental cell-fate decisions. This can occur through interaction-promoting or -inhibiting substrate modification (upper panel). Examples in which substrate phosphorylation promotes and inhibits DUB-substrate interaction include USP15-β-catenin and USP21-NANOG, respectively. Stimulus-induced phosphorylation can also occur on DUBs to promote interactions with substrates (e.g., USP9X-ankyrin G, lower panel).

DUB catalytic activity can be further controlled by reversible PTMs such as phosphorylation, ubiquitylation, SUMOylation, or oxidation [28, 59]. Regulation of DUB activity by PTMs has been shown to control physiological processes such as DNA damage responses, cell cycle progression, and innate immune signaling [26, 59]; however, little is known how this regulatory principle is employed to control development. In contrast, multiple recent studies have demonstrated that substrate recruitment to DUBs is frequently regulated by phosphorylation to ensure faithful differentiation (Fig. 2). For instance, during osteoblast differentiation, USP15 recognizes and deubiquitylates its target, the transcription factor β-catenin, only upon β-catenin phosphorylation by MEKK2 [142]. Conversely, ERK1-mediated phosphorylation of the pluripotency factor NANOG inhibits interactions with USP21, which results in proteasomal degradation of NANOG, thus facilitating rewiring of transcriptional networks during mESC differentiation [143]. In another example, USP9X undergoes TGF-β-induced phosphorylation, which does not affect its DUB activity but increases binding to its substrate ankyrin-G, resulting in ankyrin-G stabilization required for maintaining dendritic spines during neuronal differentiation [144, 145] (see further details below). Thus, during development, DUB-substrate interactions are frequently modulated by signal-induced phosphorylation, which allows DUBs to convert a particular signaling input into downstream cellular responses.

## Mechanisms how DUBs control development

**Fig. 3:**
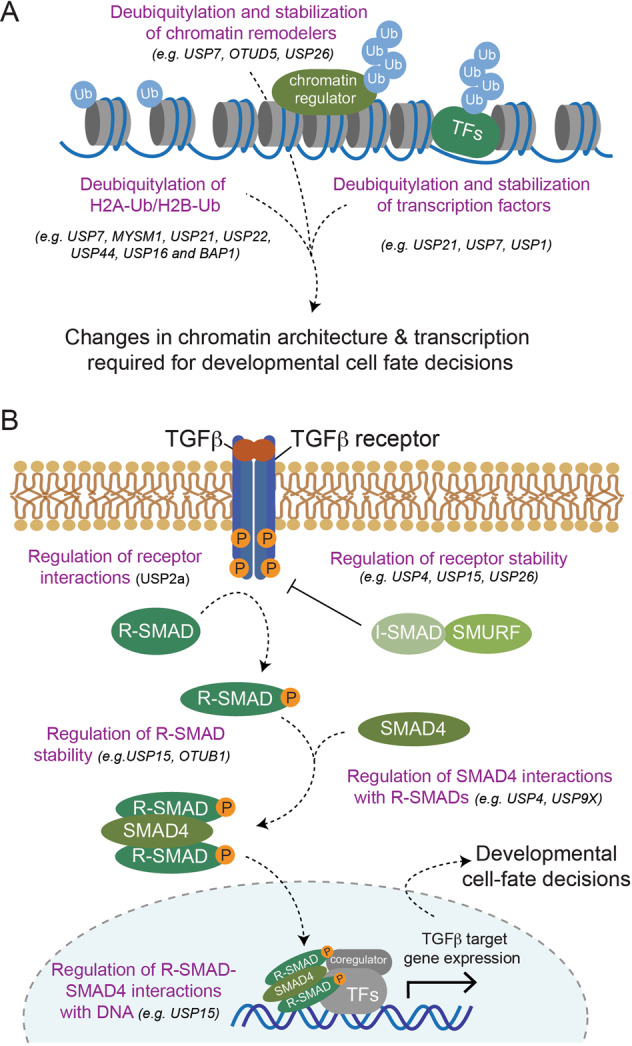
Mechanisms of how DUBs regulate ubiquitin signaling to determine developmental cell-fate decisions. **A** Schematic overview of general principles of how DUBs controls differentiation processes. Several indicated DUBs have been proposed to target ubiquitylated histones, chromatin remodeling complexes, or master transcription factors (TFs) to bring about changes in transcriptional networks required for faithful development. **B** Schematic overview of how TGF-β signaling contributes to cell-fate determination during development highlighting regulatory concepts of how DUBs modulate the strength and duration of signaling responses. Binding of TGF-β binding to the TGF-β receptor leads to receptor kinase activation and phosphorylation of receptor-activated SMADs (R-SMADs). Phosphorylated R-SMADs associate with SMAD4 to form active transcription factor complexes that translocate from the cytosol to the nucleus to elicit transcriptional responses required for developmental cell-fate decisions. This signaling cascade can be blocked by inhibitory SMADs (I-SMAD), which recruit the ubiquitin ligases SMURF1/2 (SMURF) to the TGF-β family receptors for ubiquitin-mediated degradation. Also other TGF-β signaling pathway components are subject to regulation by ubiquitylation, which is counteracted by DUBs as indicated.

Several DUBs have been shown to control different aspects of embryonic development by diverse mechanisms. This includes AMSH and USP7, which regulate endosomal sorting and membrane trafficking required for faithful neuronal differentiation [141, 146] (see below) and USP8, which maintains high levels of autophagy in mESCs required for self-renewal and pluripotency [147]. Most commonly however, DUBs regulate stem cell maintenance and differentiation by controlling gene expression (through deubiquitylating histones or through stabilizing chromatin regulators and cell-identity-defining transcription factors) or by modulating developmental signaling pathways (Fig. 3). In the following, we will outline examples for each of these mechanisms.

### DUBs regulating development through deubiquitylating histones

Dynamic changes in chromatin architecture are required for driving developmental gene expression programs. These changes are brought about by reversible histone PTMs, which alter the physical properties of chromatin and/or recruit effector proteins to alter transcription. Monoubiquitylation of H2A and H2B are an abundant and critical means for ensuring accurate gene expression during metazoan development [1, 27]. Catalyzed by a family of multi-subunit E3 ligases known as Polycomb repressive complexes (PRC1), monoubiquitylation of H2A at K119 is generally thought to silence downstream genes [148–151]. Conversely, RNF20/RNF40-mediated monoubiquitylation of H2B at K120 is generally associated with activation of gene expression through recruiting enzymes that decorate H3 with activating methylation marks [152, 153]. Several DUBs (including USP7, MYSM1, USP21, USP22, USP44, USP16, and BAP1 [79, 81, 111, 117, 143, 154–158]) have been proposed to reverse H2A and/or H2B ubiquitylation to control transcriptional networks during development (Fig. 3A). In this context, the mechanistic details of histone deubiquitylation and recruitment to chromatin have been well-characterized for only a subset of these DUBs (e.g., USP22 [107, 129, 159] and BAP1 [37, 108, 109, 111–114]). DUBs that have been reported to control developmental processes through deubiquitylating H2B include USP44, which represses genes involved in lineage commitment during mESC maintenance [79], and USP22, which specifically inhibits expression of the pluripotency factor SOX2 during hESC differentiation [81]. Examples of DUBs that are thought to elicit their functions through H2A deubiquitylation include BAP1, USP21, and USP16 [35, 37, 108, 111, 143, 160]. BAP1 and USP21 activity are required for stem cell self-renewal by ensuring the expression of genes that are involved in basic cellular functions and that are under the control of the pluripotency factor NANOG, respectively [108, 143]. In contrast, the Down Syndrome-associated USP16 is not essential for stem cell maintenance, but its activity was proposed to alleviate H2A ubiquitylation-imposed repression of lineage-specific genes during differentiation [35, 154, 160]. Thus, multiple DUBs likely cooperate to modulate chromatin accessibility and gene expression during development through counteracting H2A/H2B monoubiquitylation. In most cases, how such interplay is spatially and temporally regulated, remains to be determined.

### DUBs regulating development through controlling chromatin regulator and transcription factor stability

In addition to controlling gene expression at the level of histone deubiquitylation, DUBs also frequently target chromatin regulators and transcription factors to modulate their stability and function during stem cell maintenance and differentiation [27, 41, 161] (Fig. 3A). For example, results from somatic reprogramming studies suggest that USP26 cleaves K48-linked ubiquitin chains from the chromobox-containing proteins CBX4 and CBX6 during mESC differentiation [162]. This was proposed to stabilize these proteins and promote their function in the context of the PRC1 complex to repress the expression of pluripotency genes, ensuring faithful lineage commitment. In addition, recent reports have shown that both, USP21 and USP7 counteract degradative ubiquitylation of NANOG to ensure self-renewal of ESCs [110, 143]. This example showcases how several DUBs can target the same transcription factor and it will be interesting to further explore how such interplay regulates ESC maintenance (e.g., through targeting differently localized pools of NANOG). Conversely, the same DUB can also target several distinct transcription factors in a cell-type-specific manner. For instance, USP7, in addition to its function in maintaining hESCs [110], has been shown to control the stability of several other cell-identity-defining and lineage-promoting transcription factors, including (1) REST in neural progenitor cells to promote their maintenance [163, 164], (2) c-MYC in neural stem cells to promote their self-renewal [165], and (3) RUNX in skeletal stem cells to promote differentiation into osteoblasts [166]. Taken together, DUBs frequently target chromatin regulators or transcription factors in cell-type and tissue-specific contexts to control developmental cell-fate decisions.

### DUBs regulating development through modulating signaling pathways

Multiple signaling pathways—such as FGF, Hedgehog, WNT, TGF-β/BMP, and Notch—orchestrate development, operating repeatedly at different times and regions in the embryo to regulate germ layer specification, patterning, and organogenesis [167]. These core pathways are critically controlled by ubiquitylation and many DUBs participate in this regulation [3, 46, 168–170]. Here, to highlight concepts of how DUBs can modulate these cascades, we will focus on TGF-β/BMP signaling.

In the canonical pathway, secreted TGF-β and BMP ligands elicit their functions by activating transmembrane serine/threonine kinase receptors and intracellular second messengers known as SMADs (Fig. 3B). Binding of TGF-β/BMP ligands to their cognate receptors promotes their kinase activity, which leads to the phosphorylation of receptor-regulated SMADs (R-SMADs) [171]. Upon phosphorylation, both classes of R-SMADs associate with SMAD4 to form active transcription factor complexes that translocate from the cytoplasm to the nucleus, where they elicit downstream transcriptional responses. Finally, inhibitory SMADs such as SMAD7 are amongst TGF-β/BMP-induced genes and serve as scaffolds to recruit the ubiquitin E3 ligases SMURF1/2 to TGF-β family receptors for ubiquitin-mediated degradation. In addition to this negative feedback regulation, reversible poly- and monoubiquitylation of virtually all components of the TGF-β/BMP pathway have been shown to control the strength and duration of the signaling response. A number of DUBs participate in this regulation at multiple levels (Fig. 3B). First, several DUBs have been shown to regulate turnover of the TGF-β receptor using diverse mode of actions. This includes DUBs that stabilize the receptor by deubiquitylation of the receptor (USP4 [96] and USP15 [172]) and by deubiquitylation-dependent inactivation of SMURF2 (USP15 [173]) or DUBs that promote receptor degradation through deubiquitylating and stabilizing SMAD7 (USP26 [174]). Second, DUBs regulate protein interactions of the TGF-β receptor. This is exemplified by USP2a, which associates with the TGF-β type I and II receptors to cleave K33-linked ubiquitin chains from them, thus promoting interactions with R-SMADs and enhancing downstream signaling [175]. Third, DUBs target ubiquitylated R-SMADs to regulate their stability and interactions. For example, both, USP15 and OTUB1 counteract degradative polyubiquitylation of activated R-SMADs to promote transcriptional downstream responses [176, 177]. This occurs by different molecular mechanisms and requires catalytic activity of USP15, but not that of OTUB1, which rather binds to and inhibits the ubiquitin-conjugating activity of the cognate E2 enzyme [176, 178]. In addition, USP15 also promotes TGF-β/BMP signaling by opposing monoubiquitylation of R-SMADs, thereby allowing activated R-SMAD-SMAD4 complexes to recognize target promoters [177]. Fourth, DUBs target monoubiquitylated SMAD4 to regulate its interactions. Both, USP9X and USP4 have been shown to catalyze this reaction to promote activated R-SMAD/SMAD4 complex formation, nuclear translocation, and TGF-β-induced transcriptional activation required for zebrafish development and mESC differentiation (in case of USP4 [97]) or Xenopus development (in case of USP9X [179]).

Thus, as outlined in the above examples, multiple DUBs modulate TGF-β/BMP signaling at the receptor or effector level through prevention of degradation or control of protein–protein interactions. In this context, to achieve a certain biological outcome, the same DUB can regulate the pathway at different levels (e.g., USP15 and USP4 promote signaling by targeting the TGF-β receptor and the effector SMADs) or multiple DUBs can act on the same target (e.g., USP15 and OTUB1 promote signaling through stabilizing R-SMADs). Future experiments are required to further examine how DUB interplay at these different levels is spatially and temporally regulated to ensure proper TGF-β/BMP signaling responses during embryonic development.

## Dysregulation of DUBs results in developmental diseases

Extensive studies over the last decades have established that dysregulation of DUBs leads to human diseases, in particular cancer, neurodegeneration, and inflammation [1, 25–28, 45, 46]. In addition, mutations in DUBs frequently cause severe developmental disorders (summarized in Table 1). In general, these disorders are characterized by early-onset neurologic deficits and are thought to be caused by loss-of-function mechanisms. In the following, we will discuss select examples of DUBs that have been directly linked to monogenic developmental disorders and the proposed mechanisms of pathogenesis.

### Microcephaly-capillary malformation (MIC-CAP) syndrome (MIM:614261) caused by mutations in STAMBP/AMSH

Recessive loss-of-function mutations in *STAMBP*, also known as *AMSH*, causes MIC-CAP syndrome [146]. These patients have severe microcephaly with progressive cortical atrophy, intractable epilepsy, profound developmental delay, and multiple small capillary malformations on the skin. A variety of disease-causing mutations have been identified including frameshift, nonsense, splicing, and missense mutations, implicating loss-of-function as a mechanism of disease [146, 180–183]. Indeed, *Amsh*-deficient mice exhibit defects in cortical development similar to those in patients [39]. AMSH is a DUB that, through its K63-specific ubiquitin cleavage activity [184], controls the fate of endosomal cargos that undergo ubiquitin-dependent sorting into degradation or recycling compartments by the ESCRT pathway [65, 185]. The reported disease-causing missense mutations in AMSH are located either in the catalytic domain reducing its K63-cleavage activity [186] or in the MIT domain potentially affecting binding to components of the ESCRT pathway [146, 185]. During the pathogenesis of MIC-CAP, dysregulation of endosomal sorting likely interferes with appropriate responses to downregulate RAS/PI3K signaling, ultimately leading to the congenital anomalies observed in patients. In support of this, phenotypes of MIC-CAP syndrome closely resemble those of RASopathies, developmental disorders caused by activating mutations in the RAS-ERK signaling pathway [187]. However, the molecular details of how the loss of AMSH activity results increased RAS/PI3K signaling and the key substrates involved remain to be determined.

### Hao-Fountain syndrome (MIM:616863) caused by heterozygous mutations in USP7

*USP7* encodes an essential DUB for which disruption of one allele, whether via heterozygous deletions or nonsense/missense mutations, results in Hao-Foutain syndrome, a developmental disorder with seizures, behavioral abnormalities, hypogonadism, and hypotonia [141, 188]. Surprisingly, the molecular origin of this disease was not primarily linked to dysregulation of the many essential functions of USP7 in DNA repair, transcription, immune responses, or viral replication [189, 190], but rather to an aberrant role in cellular protein trafficking [141]. Elegant cell biological and biochemical studies demonstrated that USP7 is a component of the MAGE-L2-TRIM27 complex, a multi-subunit ubiquitin E3 ligase with well-established roles in retromer-dependent endosomal recycling of membrane proteins. MAGE-L2-TRIM27 regulates endosomal sorting through conjugation of K63-linked ubiquitin chains to WASH, thereby activating this actin nucleation promoting factor and facilitating endosomal actin assembly [191]. USP7 acts as a rheostat for this reaction by (1) deubiquitylating TRIM27 to protect it from auto-degradation and (2) by deubiquitylating WASH to limit its activity, thus fine-tuning endosomal actin assembly [141]. *MAGE-L2* is located within the Prader–Willi imprinting region [192] and was identified as the causative gene in Schaaf-Yang syndrome [193, 194]. These two disorders share many disease manifestations with Hao-Fountain syndrome, further suggesting that the *USP7*-deficiency-induced patient phenotypes are caused by aberrant endosomal sorting.

### Mental retardation, X-linked 99 (MRX99, MIM:300919, 300968) caused by mutations in USP9X

Mutations in *USP9X*, encoding an X-linked DUB, cause syndromic and non-syndromic intellectual disability. Initial studies reported three male individuals with non-syndromic X-linked intellectual disability, all carrying missense variants in *USP9X* [195]. Consistent with this, brain-specific knockout of *Usp9x* causes aberrant cortical architecture similar to that found in patients [196]. Reijinders et al. showed that heterozygous loss-of-function alleles present in females, as opposed to males, lead to a syndromic form of X-linked intellectual disability associated with characteristic facial features, short stature, cardiac, and structural brain abnormalities [197]. Together with more recent studies, this solidified a spectrum of neurodevelopmental disease in male and females with variable phenotypes, decreased penetrance, and likely variant-specific mechanisms of disease, contributing to the different sex-specific manifestations [198, 199]. USP9X is an essential DUB that, through counteracting mono- and polyubiquitylation of specific substrates, has been implicated in a plethora of cellular processes [200]. Dysregulation of several of these functions have been proposed to lead to the phenotypes observed in patients. First, as described above, USP9X regulates TGF-β signaling through deubiquitylating SMAD4 and this pathway is defective in patient fibroblasts [199]. Second, USP9X has been shown to control centriole duplication and centrosome biogenesis through e.g., deubiquitylating and stabilizing the centriole duplication factor STIL [201–203] as well as cilia assembly through regulating the localization and stability of the ciliogenesis-promoting factor NPHP5 [204]. Mutations in genes regulating these processes (including *STIL* and *ICQB1* encoding for NPHP5) frequently result in primary microcephaly [205, 206] and ciliopathies [207, 208], respectively, with considerable phenotypic overlap with *USP9X* patients, thus suggesting that aberrant centrosome duplication and cilia assembly could contribute to MRX99. Third, USP9X has been shown to regulate dendritic spine development and maintenance [144]. This occurs through deubiquitylation and stabilization of ankyrin-G, a scaffold protein that links plasma membrane proteins to the actin/β-spectrin cytoskeleton and thereby regulates multiple neurobiological processes such as synaptogenesis and synaptic plasticity [209–211]. Variants in *ANK*, encoding for ankyrin-G are associated with neurodevelopmental disorders [212] and *USP9X* patient mutations were shown to reduce interaction with ankyrin-G, strongly suggesting that abnormal ankyrin-G degradation is a pathogenic mechanism in MRX99. Consistent with this, *Usp9X* knockout mice exhibit synaptic abnormalities, ankyrin-G aggregates, and hyperactivity [144].

It is interesting to note that TGF-β promotes cortical spine development through promoting USP9X-dependent stabilization of ankyrin-G [145] and that TGF-β signaling can rely on primary cilia [213]. This raises the intriguing possibility that the aforementioned pathogenic mechanisms may be interconnected and that USP9X orchestrates neurodevelopment by acting on several distinct substrates in different pathways. Future research should focus on such interplay and test the relative contributions of different substrates and functions to the sex-specific MRX99 manifestations.

### Intellectual developmental disorder with dysmorphic faces, seizures, and distal limb anomalies (MIM:617452) caused by recessive mutations in OTUD6B

Bi-allelic loss-of-function of *OTUD6B* causes global developmental delay, feeding difficulties, structural brain abnormalities, and congenital heart disease [40, 214]. OTUD6B is a poorly characterized OTU DUB with no clearly assigned in vitro deubiquitylation activity or ubiquitin-linkage preference [61]. It has been connected to protein translation [215] and may regulate proteasome stability [40]; however, further mechanistic studies are required to establish whether loss of these or other functions of OTUD6B drive the aberrant differentiation processes observed in *OTUD6B* patients.

### 15q13.3 microdeletion syndrome (MIM:612001) caused in part by haploinsufficiency of OTUD7A

*OTUD7A*, encoding a poorly studied K11-specfic OTU DUB [61], is located in the 15q13.3 locus, which when deleted causes a wide spectrum of neurodevelopmental and psychiatric disorders [216–219]. 15q13.3 microdeletion syndrome is the most common genetic cause of epilepsy [220]. Recent studies have shown that out of the six protein-coding genes that are typically encompassed in the deletions, *OTUD7A* is the most likely candidate to cause associated epilepsy. First, studies in mice have shown that OTUD7A controls dendritic branching of cortical neurons [86]. Second, knockout of *Otud7a* recapitulated neurodevelopmental deficits including abnormal EEGs [87]. Third, an individual with neurodevelopmental phenotypes and epilepsy carrying biallelic *OTUD7A* missense variants has been reported [221]. These findings highlight an important role in OTUD7A in controlling neurodevelopment; yet, the molecular underpinnings of this regulation, including cellular mechanisms and cognate E3 ligases and substrates, have remained largely unclear. Their identification will have important implications for understanding distinct forms of epilepsy.

### Linkage-specific deubiquitylation deficiency-induced embryonic defect (LINKED) syndrome caused by mutations in OTUD5

Hemizygous missense and deletion variants in *OTUD5*, encoding an X-linked OTU DUB that prefers cleavage of K48- and K63-linked ubiquitin chains [61, 222–224], have recently been shown to cause a male-specific multiple congenital disorder [41]. Affected patients suffer from a spectrum of central nervous system, craniofacial, cardiac, skeletal, and genitourinary anomalies. OTUD5 has previously been implicated in regulating innate and adaptive immune signaling [224–226]; however, the reported patient phenotypes suggest an additional role of this enzyme during embryonic cell-fate determination. Indeed, knockout of *Otud5* is embryonic lethal in mice and OTUD5-depleted hESCs are defective in neuroectodermal differentiation, which can be rescued by re-expression of wild-type OTUD5 [41]. Interestingly, a patient variant that affects K48- but not K63-ubiquitin chain cleavage activity, is not able to rescue the differentiation defects, suggesting that the disease originates from loss of OTUD5’s activity towards degradative K48-linked ubiquitin chains. Corroborating this notion, OTUD5 prevents the degradation of multiple chromatin remodelers to coordinate enhancer activation during neuroectodermal differentiation. Amongst these OTUD5 substrates are ARID1A/B, HDAC2, and HCF1, mutations of which underlie different developmental disorders (Coffin–Siris and Cornelia de Lange syndromes [227, 228], X-linked mental retardation 3 [229]) that exhibit considerable phenotypic overlap with LINKED patients. Thus, this work reveals K48-ubiquitin chain cleavage of functionally related substrates as an essential signaling mode coordinating chromatin remodeling during early human development. Additional experiments are required to determine the molecular details of this regulation in the broader context of embryogenesis.

## Conclusion and perspectives

**Fig. 4:**
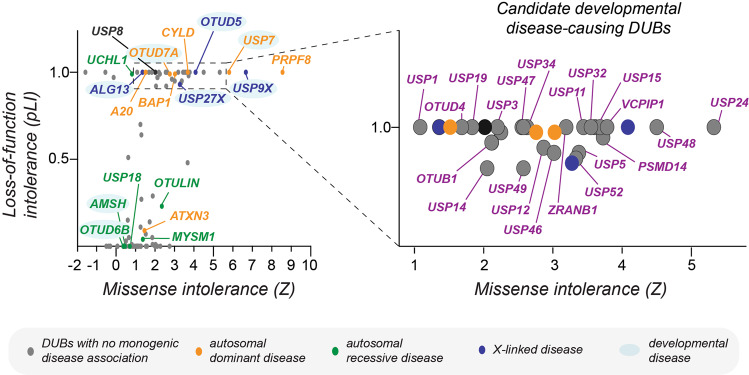
Many DUBs are intolerant to genomic variation in humans and are likely to cause developmental disease when mutated. Graph depicting a plot of missense (Z) and loss-of-function intolerance (pLI) scores of all human DUBs (as determined using gnomAD [233]). Highlighted in color are DUBs whose mutations have been demonstrated to cause monogenic diseases that are inherited in an autosomal dominant manner (orange), autosomal recessive manner (green), or X-linked dominant/recessive manner (blue). Mutations in USP8 (highlighted in black) cause corticotroph adenomas and Cushing’s disease in the somatic state. Note that DUBs associated with autosomal dominant and X-linked disease are constrained in their genomic variation within the healthy human population (pLI ≈ 1, *Z*-score > 1). Many other DUBs, previously not linked to monogenic diseases, are also highly intolerant to missense and loss-of-function mutations and thus likely cause embryonic lethality or developmental disease when mutated. The strongest of these candidates are highlighted in violet in the zoomed-in panel of the plot on the right.

Since the initial discovery of DUBs almost 40 years ago, numerous studies have provided insights into their structures, substrate/cleavage specificities, and regulatory mechanisms that allow this versatile enzyme family to contribute to diverse cellular processes. In particular, we here highlight principles of how DUBs modulate ubiquitin signaling during embryonic and postnatal development and the emerging roles of their dysregulation in congenital disorders. Despite many recent advances in our understanding of DUBs in these (patho-)physiological processes, many open questions remain. First, for more than half of the human DUBs, substrates and linkage specificities have remained unclear [26]. Moreover, as several DUBs are relatively large proteins challenging to produce in bacteria, many biochemical activities have been determined with truncation variants, which could lack important specificity determinants encoded in the full-length protein. Similarly, as detailed in this review, PTMs and co-factors have been shown to regulate DUB activity and linkage-specificities in cells and those contributions are not captured during in vitro activity assays using bacterial proteins. Therefore, characterizing DUB mechanisms and specificities by in vitro and cell-based assays, particularly focusing on full-length proteins, will be important to further define physiological roles of DUBs and elucidate their role in disease. Second, as alluded to throughout this review, it is often unclear how the intricate regulatory mechanisms that can regulate DUB localization, activity, and substrate recruitment in vitro are implemented to ensure faithful embryonic and postnatal development in vivo. Third, while mutations in ~10 DUBs have been convincingly demonstrated to cause developmental disease (Fig. 4), the underlying mechanisms, E3 ligases, and/or substrates are often ill-defined (e.g., *OTUD6B* and *OTUD7A*). Fourth, knockout or knockdown of tens of DUBs has been shown to be lethal or to cause severe defects during embryogenesis of model organisms such as zebrafish and mice [43, 44]. In many cases, these DUBs have not yet been associated with congenital disorders and/or their precise functions and underlying mechanisms in early human development are not known (e.g., *OTUD4*, *USP25*).

With the rapid increase in databases of exome and genome sequences from healthy individuals, it has now become possible to quantify the tolerance of genes to loss-of-function and missense mutations in control populations. [230–233] Genes that are highly restricted in such variation are likely to be essential and, when mutated, either result in embryonic lethality or developmental disease. As recently demonstrated for *OTUD5* and LINKED syndrome [41], such genomic constraint metrics can be used to prioritize candidate disease variants and, combined with mechanistic studies, facilitate the discovery of novel developmental pathways. Intriguingly, there are many DUBs, not yet associated with congenital disorders, but that are likely to be disease-causing based on how constrained they are from mutations in the healthy population (highlighted in violet in Fig. 4). We propose that systematic search for missense variants in these genes in patients with undiagnosed diseases, will likely allow identification of novel developmental disorders and may yield variants that can be used to dissect functions and mechanisms of these DUBs during embryogenesis. Even if such patients are not readily identified, these tools provide clues about enzymes important for human health to prioritize for mechanistic studies. Such genomic constraint-based genotype-first approaches would be especially interesting for poorly characterized DUBs such as *USP24*, *USP48*, and *USP32*. It would be equally attractive to apply this methodology to the linkage-specific OTU DUBs *OTUD4*, *OTUB1*, *VCPIP*, and *ZRANB1* [61] to uncover potentially novel roles of particular ubiquitin chain types during early development. Finally, such methodologies could provide important mechanistic insights to help improve disease diagnosis and patient management and, given the growing ability to target the activity of specific DUBs with small molecules [26, 45, 234], potentially open new avenues for therapeutic intervention to ameliorate disease symptoms.
